# On Inflammatory and Spasmodic Strictures of the Urethra

**Published:** 1834-01-01

**Authors:** Fredk. Tyrrell

**Affiliations:** 17, New Bridge street, Blackfriars


*To the Editor of the Medical Quarterly Review.*

Sir: According to the intention expressed when I last had the pleasure of addressing you, I now forward the result of my observations on Inflammatory and Spasmodic Strictures of the Urethra; and have the honour to remain,

Yours respectfully,

Fred[K]. Tyrrell.

17, *New Bridge street, Blackfriars;*

*December 23d,* 1833.

*Of Inflammatory Stricture.*

By the term inflammatory stricture, I understand a narrowing or closure of the urethra, in consequence of acute disease of the mucous membrane and adjacent parts, which must occur in a degree whenever acute inflammation exists in these structures.

*Symptoms.* Pain and tenderness along the urethra, usually, however, confined more particularly to some portion of the passage. A frequent desire to void the urine, which is ejected in a small, twisted, or scattering stream, and attended with a sense of heat or scalding in the canal, especially at that part where the tenderness is greatest. Sometimes the stream is so diminished as not to exceed a small thread in diameter, but I have not known an instance of perfect closure of the urethra from inflammation without spasm.

*Causes.* It is generally a consequence of gonorrhœa, but is also produced by injury. In the former instance the disease may be confined to the ordinary seat of gonorrhœal inflammation, about one inch and a half or two inches from the meatus, or it may extend nearly throughout the canal. In the latter case the affection is usually confined to the seat of injury.

*Proximate Cause.* The condition of the diseased part I believe to be similar to that state of the conjunctiva termed chemosis, or when it is thickened and elevated, by distention of its vessels, and by deposition of serum and fibrin in the subjacent cellular tissue.

*Treatment.* The patient should be kept in the recumbent posture, and the genitals should be well supported towards the abdomen. Leeches should be applied along the under part of the urethra, and subsequently fomentations and poultices. The hip-bath is often very serviceable; and, after the application of leeches, subjects the patient to less inconvenience and annoyance than the fomentations. The bowels should be freely acted on by some mercurial, followed by an alkaline salt in solution. The patient should drink sparingly of some mucilaginous liquid. The diet should be light, and consist principally of milk with farinaceous substances. If there be much irritability of the bladder, a suppository or opiate injection into the rectum may be advantageously used, when the bowels have been freely opened.

In some cases, when the local symptoms are severe, and there is much constitutional disturbance of an acute kind, it may be requisite, in the commencement of the treatment, to abstract blood from the arm.

Perseverance in these means will speedily subdue the acute disease, but the surgeon must be cautious not to neglect his patient until he is satisfied that the effects of the inflammation are annihilated; otherwise, the formation of permanent stricture is very likely to ensue: he should therefore observe the stream of urine, and examine the course of the urethra, to learn whether any deviation from the natural stream still exists, or whether there be any tenderness or thickening along the canal.

If simply a slight irregularity in the stream of urine remains, with trifling tenderness in some part of the urethra, these symptoms will gradually subside if the patient remains quiet, keeps his secretions in good order, and avoids stimulating food and drink. But, should there be a very small or irregular stream of urine, with some hardness as well as tenderness about the urethra, and uneasiness during erection, something further must be done.

The most effectual plan is that of applying a blister over the seat of the thickening of the canal; it should be allowed to remain on for sixteen or eighteen hours, and, when removed, a light poultice of bread and water should be used until the part has healed. Otherwise, some stimulating ointment may be rubbed into the part night and morning; such as the blue ointment with camphor, or the ointment of the hydriodate of potash.

When the thickening about the urethra is considerable, I should advise the use of mercury, (provided there be a state of general health admitting it,) so as to affect the system slightly; having seen much advantage from it in expediting the absorption of the morbid deposit.

This condition of the urethra I alluded to in my observations on Permanent Stricture, as being so frequent after gonorrhœa, but which can, I believe, always be subdued by careful treatment, without the aid of mechanical means, as bougies, catheters, &c. Such means should never, in my opinion, be resorted to until the plan I have described has been fully and fairly tried without success.

*Of Spasmodic Stricture.*

It consists in a partial or complete closure of the urethra by involuntary muscular contraction.

It may take place without any evidence of previous disease in the canal, or it may occur during the existence of permanent or inflammatory stricture.

*Symptoms.* In the first instance, the patient will experience a sudden interruption or obstruction to the flow of urine, attended with uneasiness or pain in the region of the perineum, and frequent and urgent desire to void the urine.

If the patient has previously suffered from permanent stricture, he may find a sudden inability to discharge the urine, accompanied with the above symptoms, only generally of more severe character.

If spasm occurs during the existence of inflammatory stricture, the retention of urine is accompanied with excessive suffering; the pain in the perineum, and in the course of the urethra, generally being excessive, and the part very tender on pressure.

If the spasm closes the urethra completely, and occasions retention of urine, there is usually constitutional disturbance, evinced first by rigors, and afterwards by febrile action proportioned to the severity of the local affection; greater, therefore, when the spasm is combined with inflammatory stricture, than when there has not been previous disease of the urethra.

*Causes.* Exposure to cold and damp, or the imprudent or excessive use of spirits. The influence of these causes may be noticed in almost any case of permanent stricture, when the stream of urine always becomes lessened in damp and cold weather, or in consequence of any debauch in wine or spirits.

*Predisposing Causes.* Acute or chronic disease of the mucous membrane of the urethra.

*Proximate Cause,* I consider to be partial or complete spasmodic contraction of the accelerator urinæ muscle.

The most attentive and careful examination of the urethra and its parietes does not exhibit any trace of muscular fibre in contact, or nearly so, with the mucous membrane, as some have supposed. The only muscular structures which can exert any influence over the diameter of the canal are, first, that denominated the accelerator urinæ, or ejaculator seminis; and, secondly, a portion of the levator ani, described by Mr. Wilson as distinct muscles, under the names of levatores urethræ.

The former, the accelerator urinæ, is attached superiorly and posteriorly to the under surface of the triangular fascia which occupies the pubic arch, and inferiorly and anteriorly it is connected by muscular slips to the corpora cavernosa. Between these attachments the muscle covers the corpus spongiosum (that part called the bulb,) to the extent of an inch or more; the muscular fibres being united beneath the corpus spongiosum on the median line, from whence they are continued forwards and upwards, so as to embrace the corpus spongiosum inferiorly and laterally, and become united to a tendon which is placed between the corpus spongiosum and corpora cavernosa, so that the muscle and tendon together completely surround the bulbous part of the spongy body; the muscle is consequently capable of closing the urethra by its actions.

The levatores urethræ I have not been able, by most careful dissection, to expose as described by Mr. Wilson; but, even allowing his description to be correct, the muscles only support the membranous part of the urethra as a sling, and could not, by their most forcible action, afford that resistance which we meet with in spasmodic stricture.

There being therefore no structure but that known as the accelerator urinæ, which is capable by its action of closing the canal, our attention would be naturally led to the situation of this muscle when spasm exists; and my own experience (which has been considerable,) has satisfied me that the seat of spasmodic stricture is always in that part of the urethra enclosed by the bulb of the corpus spongiosum, and by the accelerator muscle.

I have stated, in my former observations on Stricture, how readily spasm is induced in this part of the canal by the irritation of bougies or other instruments, but which does not occur in any other situation. This is a proof of the influence of the muscle.

*Treatment.* Surgical assistance is not generally sought for unless the spasmodic action be sufficient to produce retention of urine; for the partial spasm is never of long duration, or productive of any serious inconvenience. I shall therefore only consider the treatment as applicable to the condition of perfect retention: 1st, as resulting from purely spasmodic stricture; and, secondly, when the spasm occurs during the existence of other disease in the urethra.

When retention of urine takes place with the symptoms I have described as indicating spasm, there not having been any evidence of previous disease in the passage, the patient may be relieved in most instances by simple means: but the disease becomes aggravated under severe local treatment. The surgeon should first endeavour to ascertain the cause of the urethral affection, and the amount of general disturbance.

If it has resulted from exposure to cold and damp, there will generally be a sensation of universal chilliness and rigors. The patient should then be placed in a warm bed, and should take a full dose of opiate combined with some mercurial, and have a general or hip bath as soon as convenient, at a temperature of ninety-eight or one hundred degrees; otherwise, the perineum and lower part of the abdomen should be well fomented. If the patient is not relieved by these means, the surgeon should administer some purgative, either by mouth or by injection; the latter mode being generally preferable, as producing the most speedy effect. The enema may consist of some senna and salts, or gruel, with the sulphate of magnesia or castor oil; and, after the bowels have been relieved, opium may be again given. Some surgeons recommend that the tinctura ferri muriatis should be administered with the opium, in the dose of thirty or forty minims. I have frequently seen it employed, and I think in some cases with decided advantage.

It is generally allowed that mechanical remedies are improper and injurious at the commencement of the treatment; and I perfectly agree in this opinion, but would strongly recommend a local remedy which I have found most beneficial, and consider likely to afford relief in a large majority of cases: it is the application of the extract of belladonna to the seat of stricture. I first used this extract in the winter of 1819, and have had numerous opportunities of employing it since, and am perfectly satisfied of its being a very valuable remedy. The mode of application is as follows: a common wax bougie, of moderate size, should have the rounded extremity cut off, and a small excavation made at the end. This small cavity is to be filled with the extract of belladonna, and the bougie is also to be smeared with the extract mixed with oil. The instrument is then carefully and gently to be passed into the urethra until the extremity, loaded with belladonna, reaches the seat of stricture; it should then be pressed against the part with a very moderate force, and kept there for ten or twelve minutes, or until the patient expresses a wish to void urine. I have generally, found that the patient has been able to discharge urine when the bougie has been withdrawn; but I have sometimes been obliged to introduce a bougie or catheter before the patient could micturate; and this I have effected usually with facility after the application of the belladonna in the way I have described.

In the greater number of cases in which I have employed this remedy it has been, after repeated trials and failures of the ordinary mechanical means, and where these means have produced injury to the mucous membrane, indicated by considerable hæmorrhage; but even in these cases it has been successful.

During the last year, a man was admitted into the accident ward of St. Thomas's Hospital, with retention of urine from purely spasmodic stricture, after exposure to cold, having within twelve hours before his admission passed his urine in a full and free stream. He was about thirty years of age, of spare habit, and not addicted to the use of spirits. My dresser tried the effect of general bloodletting and the warm bath, which produced syncope, but did not relieve the spasm. He then resorted to the bougie and catheter; but, failing to pass them through the stricture, he sent for me. On my arrival at the hospital, I found the man very feeble, with his bladder greatly distended, and bleeding from the urethra, which was also extremely tender, from the force which had been employed in endeavouring to introduce the instruments. I immediately applied the belladonna in the way I have described, keeping the bougie pressed lightly against the stricture for about twelve minutes, when the patient said that he felt as if he could pass his urine if I withdrew the instrument. In this, however, he was disappointed; and I then introduced a middle-sized common wax bougie smeared with oil in the usual way, and with scarcely any difficulty passed it into the bladder. I allowed it to remain in about a minute, and, directly I withdrew it, the patient voided urine freely.

The preceding year a man came to the surgery in the hospital, having retention of urine from spasmodic stricture, after drinking more freely than usual of spirits. The gentleman then on duty as dresser, who had seen me use the belladonna with good effect, immediately applied it in this case, and succeeded in relieving the man in the course of five or six minutes, without the aid of any other remedy. The man having obtained relief so speedily, and with scarcely any suffering, in three or four days afterwards drank largely again of spirits, and again had retention of urine, for which he sought relief at the hospital. Fortunately, the same dresser was on duty, and, by the application of belladonna, relieved the case as speedily as before. This patient came a third time, some weeks afterwards, under similar circumstances, when the belladonna was once more applied with equally good effect.

The surgeon may not be able always to procure this extract, and may not succeed in overcoming the spasm by bleeding, the warm bath, opiates, purgatives, or other general means, when he must resort to some local treatment. There are two plans which I have known succeed; the first has been the introduction of an elastic gum bougie, or catheter, which has been very carefully passed as far as the stricture, and then pressed against it with moderate force for several minutes; after which the stricture has yielded, and admitted the instrument into the bladder. A common catheter may answer the purpose; but I consider the kind of instrument least likely to produce irritation the best calculated to succeed. The pressure employed should be firm, but still not sufficient to risk the laceration of the mucous membrane. The other plan has been the use of the nitrate of silver. The extremity of a wax bougie being armed with a minute piece of the caustic, nearly in the same way as I have directed the belladonna to be applied, has been introduced into the urethra, so as to carry the nitrate of silver to the stricture. The caustic being allowed to remain in contact with the affected part for a few seconds, has been withdrawn; and, if the patient has not then been able to discharge urine, a common bougie or catheter has been used. But I have known both plans fail, and the belladonna to succeed after such failure.

In cases of purely spasmodic stricture, I do not see any decided objection to the application of belladonna before other remedies; but consider its operation likely to be facilitated by the previous employment of the general means before mentioned.

When there is evidence of previous disease of an acute kind in the urethra, the general treatment is absolutely necessary, as there is little probability of overcoming the spasm whilst the local action continues severe. I should therefore recommend general and local bloodletting, the warm bath, purgatives, followed by narcotics, so long as the degree of pain and the extent of tenderness about the urethra indicate the continuance of acute inflammation. These means generally subdue spasmodic action; but, should they fail, I should resort to the belladonna as before described.

The combination of spasmodic with permanent stricture is a very frequent occurrence, so much so, as to be the cause of retention of urine in more than one half of the cases in which such affection takes place.

The morbid change which constitutes permanent thickening may, as I have described in my former communication, so far reduce the calibre of the urethra as to permit the urine only to pass guttatim; but this is rarely the case, for the patient usually seeks advice long before the disease has reached this extent, or is compelled to do so in consequence of sudden retention.

Supposing a person to be the subject of permanent stricture, which has produced great alteration in the stream of urine, reducing it to the diameter of a coarse thread, and that, having voided the urine in a stream of such size in the morning, he becomes unable in the course of a few hours to discharge the smallest quantity, or that he suffers from retention: in such a case the retention does not arise from the permanent stricture alone, but from spasm.

The progress of the chronic affection which produces permanent stricture never proceeds so rapidly as to close the canal in a few hours, but retention frequently takes place in such cases. When therefore a patient has been able to pass his urine even in a very fine stream within twelve hours of the period of retention, I believe the retention to be occasioned by spasm; and, with this conviction, should treat the patient, as in the former instances, by warm bath, local bleeding, purgatives, and opium at first; and then by the application of the belladonna. The first case in which I had an opportunity of employing the remedy, was of the kind last described. The patient was between fifty and sixty years of age; had been for years the subject of permanent stricture, which had reduced the stream of urine to the size of a small crow-quill. Retention occurred after drinking spirits. I was called to see him in consequence, his general medical attendant having failed to relieve him by purgatives, hip-bath, and catheter: the latter means had caused free hemorrhage from the urethra. I had gone provided with belladonna, having for some time been anxious to try it. I immediately applied it with a bougie, and in the course of a few minutes the gentleman said he felt as if he could discharge the urine, which he did as soon as I withdrew the bougie.

I have since had very frequent opportunities of trying the belladonna in somewhat similar cases, and in a large majority with equally good effect. A gentleman, who consulted me some years since, being the subject of permanent stricture, which had reduced the stream of urine to the size of a small straw, but who would not submit to treatment for the removal of the chronic thickening, has been attacked with retention at least half a dozen times in the course of the last three years, and has been each time relieved by the warm bath, opium, and belladonna. The two former remedies have always been tried before the belladonna, so that the relief could be attributed to their influence alone.

Most of those gentlemen who have acted as dressers under me at St. Thomas's Hospital have witnessed the good effects of this remedy for spasmodic stricture: indeed, within the last week, I was called to a case of retention at St. Thomas's Hospital, after the warm bath and the catheter had been ineffectually employed. I first applied the extract of belladonna, and, after five or six minutes, passed a small-sized wax bougie into the bladder, without the slightest difficulty.

In the foregoing observations I have two objects in view: 1st, to get rid, as far as possible, of mechanical means for the relief of retention from stricture, being convinced that there are very few cases to which such means are properly applicable; and, 2dly, to make known more generally a remedy which has, in my own practice, proved of the greatest value.

